# Acute Brucellosis Presenting with Bleeding Tendency due to Isolated Severe Thrombocytopenia

**DOI:** 10.1155/2018/7867435

**Published:** 2018-09-23

**Authors:** M. Aon, T. Al-Enezi

**Affiliations:** ^1^Department of Medicine, Faculty of Medicine, Cairo University, Giza, Egypt; ^2^Department of Medicine, Jahra Hospital, Al-Jahra, Kuwait

## Abstract

Mild anemia and leukopenia are the most common hematologic findings in the course of acute brucellosis. Severe form of thrombocytopenia is less frequently reported. We describe a case of acute brucellosis in a 20-year-old man, who presented with fever, purpuric skin lesions, epistaxis, and hematuria. The absolute platelet count was 2 × 10^9^/L. The patient was diagnosed as suffering from brucellosis on the basis of a strongly positive serologic reaction and was treated with antibiotics and a short course of corticosteroids, with a rapid rise in platelet count. *Brucella* infection can cause immune-mediated thrombocytopenia that is reversible after appropriate antimicrobial therapy and steroid treatment.

## 1. Introduction

Brucellosis is one of the most important and widespread zoonoses in the world. In humans, brucellosis can be caused by *B. melitensis*, *B. abortus*, *B. suis*, and *B. canis* [1]. Mild hematological abnormalities, such as anemia and leukopenia, are common in the course of human brucellosis; however, they generally resolve with treatment of the disease. Thrombocytopenia is less common, and it is rarely severe enough to cause bleeding [2, 3]. Hereby, we describe an acute brucellosis patient who presented with bleeding manifestations (epistaxis, gingival bleeding, and hematuria) and severe thrombocytopenia consistent with immune thrombocytopenic purpura.

## 2. Case Report

A 20-year-old male patient with no past medical history presented to the emergency room (at Jahra Hospital, Kuwait) with history of fever associated with malaise and arthralgia for 7 days. On arrival in the causality, he was febrile (39°C). Rest of the physical examination was normal. A history of ingesting unpasteurized camel's milk was reported, and a serological test for brucellosis was positive. A complete blood count (CBC) showed hemoglobin 145 g/L, WBCs 7.6 × 10^9^/L with 45.6% lymphocytes and 49.2% neutrophils, and platelets 4 × 10^**9**^/L. Other laboratory tests showed urea 3.6 mmol/L, creatinine 75.25 *µ*mol/L, total bilirubin 16.1 *µ*mol/L, ALT 48 IU/L, and AST 52 IU/L. There was severe isolated thrombocytopenia (4000), but no evidence of skin or mucous membranes bleeding. He denied use of any over-the-counter drugs, painkillers, or NSAIDs. The ER physician advised for hospital admission but he refused. Therapy with doxycycline and rifampicin was prescribed, but then he left against medical advice. Two days later, he returned to the emergency room with complaints of epistaxis, gingival bleeding, and hematuria. No hematemesis or melena was reported. He was febrile with a body temperature of 38.7°C, an arterial blood pressure of 110/75 mmHg, and a heart rate of 72 beats/min. There was nonitchy flat purpuric rash, particularly on his lower extremities. His physical examinations of the cardiovascular, respiratory, abdominal, and central nervous systems were normal. Repeat CBC showed hemoglobin 128 g/L, WBCs 4.9 × 10^9^/L with 51.8% lymphocytes and 41.1% neutrophils, and platelets 2 × 10^9^/L. A peripheral blood smear examination showed normal RBCs, WBCs morphology, and marked thrombocytopenia with occasional giant platelets and no platelet clumps. When true thrombocytopenia was confirmed and to exclude other causes of thrombocytopenia, malaria blood film, coagulation profile, fibrinogen level, antinuclear antibody (ANA), double-stranded DNA antibody (anti-ds DNA), and serological tests for HbsAg, anti-HCV, HIV, cytomegalovirus, and Ebstein–Barr virus all were unremarkable. *Brucella* agglutination test was positive (titer 1 : 1280). We considered a diagnosis of brucellosis with immune thrombocytopenic purpura. Due to the very low platelet count and the mucous membrane hemorrhage, 12 units of platelets were infused. Treatment with intravenous immunoglobulin (IVIg) was initiated at a dose of 500 mg/kg/day, combined with prednisolone (1 mg/kg/day), doxycycline (100 mg/12 hours), and rifampicin (600 mg/day). Fever resolved on the second day of treatment, platelet count started to rise, and bleeding manifestations improved. On the 6^th^ day, his platelet count was 66000, and he was discharged from hospital on tablet prednisolone of 25 mg daily with gradual tapering, tablet rifampin of 600 mg daily, and capsule doxycycline of 100 mg twice a day for total six weeks. Blood cultures performed on specimens obtained at the time of admission showed no growth. He was planned for follow-up one month after discharge from the hospital, but he did not show up. A follow-up phone call confirmed that all his symptoms resolved and he finished his treatment course.

## 3. Discussion

Brucellosis is a chronic granulomatous infection caused by Gram-negative coccobacilli of the genus *Brucella* and transmitted to humans through direct contact with infected animals or through consumption of unpasteurized dairy products [4]. Human brucellosis remains the commonest zoonotic disease worldwide with more than 500,000 new cases annually. The annual incidence in Kuwait is 33.9 cases per million. Despite the developing economy and advancing technology, consumption of raw milk products is still common and the region is considered an endemic area [5].

Most of the *Brucella* patients (44.8–60.5%) have a history of raising livestock or occupations carrying a risk for brucellosis. Consumption history of dried raw meat or dairy products is present in 24.6–35.1% of cases [3, 6]. The study case was infected through drinking unpasteurized milk. Studies showed that raw milk consumption is the commonest route of infection in Kuwait [7, 8].

Human brucellosis is a disease of protean manifestations. The most frequent symptoms are fever, malaise, sweating, arthralgia, headache, and back pain. Physical examination may reveal fever, hepatomegaly, or splenomegaly. *Brucella* infection may involve any organ or tissue in the body, e.g., the locomotor, genitourinary, gastrointestinal, hematological, cardiovascular, respiratory, and central nervous systems [6].

Date from the largest case series in the literature showed that the most common laboratory findings were high CRP levels (58.4%), high ESR (51.3%), anemia (40.3%), lymphocytosis (28.2%), and transaminase elevation (24.8%). The standard tube agglutination test was positive in 94.1% of cases [3].

In brucellosis, the blood count is often characterized by mild leukopenia and relative lymphocytosis along with anemia [9]. Although laboratory abnormalities with regard to the hematological system are common in the course of human brucellosis, isolated thrombocytopenia is less common (1–8%), and it is rarely severe enough to cause bleeding [2, 10].

Isolated thrombocytopenia complicating *Brucella* infection results from multiple possible mechanisms. Among the proposed mechanisms are hypersplenism, bone marrow suppression, disseminated intravascular coagulation, and hemophagocytosis [11]. Immune-mediated thrombocytopenia, which has not received major attention, is an important mechanism encountered during the course of brucellosis. An immune response is activated in the course of brucellosis which may cause an autoimmune hemolysis and destruction of platelets. Immune thrombocyte destruction may significantly threaten life due to severe bleeding [12].

The main mechanism of thrombocytopenia in our patient was possibly related to brucellosis-induced antibodies reacting with thrombocytes. Normal coagulation profile, normal fibrinogen level, and absence of schistocytes in the peripheral blood film were against DIC. There was no splenomegaly, so we cannot refer the thrombocytopenia to splenomegaly and hypersplenism. There was no evidence of bone marrow suppression as WBCs, RBCs, and reticulocytes were all within the normal limit. Bone marrow suppression and hypersplenism are usually associated with pancytopenia. In the study case, only isolated thrombocytopenia was observed. To exclude other causes of thrombocytopenia, the virology panel, malaria blood film, ANA, and anti-ds DNA were tested and results came negative.

Evidence for an immune mechanism may include rapid response to corticosteroids, positive Coombs tests, or the presence of antiplatelet antibodies. Antiplatelet antibodies can be difficult to detect by the usual tests and have a little correlation between their titer and the degree of thrombocytopenia. A negative assay for antiplatelet antibodies does not exclude the diagnosis of immune thrombocytopenia [10].

Combining therapies (corticosteroids and IVIg) is appropriate for the emergency treatment of primary immune thrombocytopenia patients with uncontrolled bleeding and severe thrombocytopenia especially when an urgent increase in platelet count is required, e.g., before surgical procedures or with active central nervous system (CNS), gastrointestinal (GI), or genitourinary bleeding [13].

In brucellosis, thrombocyte recovery usually occurs within 1–3 weeks of initiating appropriate antimicrobial therapy. However, in cases of severe thrombocytopenia, an urgent treatment institution of glucocorticoids will result in an increased platelet count and can be used to control bleeding until the antimicrobial therapy takes effect [14]. If a patient has mild-to-moderate thrombocytopenia with no life-threatening bleeding, antibrucellosis treatment should be started only. It is suggested that corticosteroids should be started along with antibrucellosis treatment in cases of which platelet count is <10 000 [11].

Although we initiated treatment with IVIg and steroids, we believe that, in most cases of *Brucella*-associated thrombocytopenia, treatment with corticosteroids along with antibrucellosis treatment will suffice. The rapid response to treatment supports the notion that immune-mediated one is the culprit mechanism.

The percentage of cases with positive cultures ranges from 11.4 to 38. Serology is the preferred method for the diagnosis of brucellosis when bacterial isolation is not possible. Titers ≥1 : 160 are considered diagnostic in conjunction with a compatible clinical presentation. In endemic areas, using a titer ≥1 : 320 may be more specific [3, 15].

In conclusion, brucellosis should always be kept in mind for the differential diagnosis of isolated thrombocytopenia in endemic areas. Patients should be investigated for brucellosis in the presence of fever or a travelling history to countries where the disease is endemic. Although it is rare, if diagnosed properly, dramatic response to the treatment is expected.
